# Growth Assessment and Nutritional Status in Children with Congenital Adrenal Hyperplasia—A Cross-Sectional Study from a Vietnamese Tertiary Pediatric Center

**DOI:** 10.3390/diagnostics15121534

**Published:** 2025-06-16

**Authors:** Thi Thuy Hong Nguyen, Khanh Minh Le, Thi Anh Thuong Tran, Khanh Ngoc Nguyen, Thi Bich Ngoc Can, Phuong Thao Bui, Dat Tien Tran, Chi Dung Vu

**Affiliations:** 1 Department of Paediatrics, Hanoi Medical University, Hanoi 11521, Vietnam; bshong@hmu.edu.vn (T.T.H.N.); lekhanhminhmj111@gmail.com (K.M.L.); trananhthuong@hmu.edu.vn (T.A.T.T.); khanhnn@nch.gov.vn (K.N.N.); ngocctb@nch.gov.vn (T.B.N.C.); trantiendat1008@gmail.com (D.T.T.); 2 Center for Endocrinology, Metabolism, Genetic/Genomics and Molecular Therapy, Vietnam National Children’s Hospital, Hanoi 11512, Vietnam; thaobp@nch.gov.vn

**Keywords:** congenital adrenal hyperplasia, CAH, growth, bone age, obesity, vitamin D deficiency, calcium deficiency, Vietnamese children, glucocorticoid treatment

## Abstract

**Background/Objectives:** Children with congenital adrenal hyperplasia (CAH) face significant risks of impaired growth and metabolic disturbances despite standard glucocorticoid therapy. This cross-sectional study aimed to evaluate growth outcomes, nutritional status, and associated factors among children with CAH treated in a Vietnamese tertiary pediatric center. **Methods:** We assessed 201 children aged 1.1–16.5 years in a tertiary pediatric center in Vietnam for anthropometric parameters, biochemical markers (calcium, phosphate, 25-hydroxyvitamin D), and clinical features. Growth status was evaluated using WHO standards, and bone age was assessed radiographically. Statistical analyses explored associations between growth outcomes and clinical, biochemical, and treatment-related factors. **Results**: Stunting was present in 16.4% of children, while 53.3% were overweight or obese. Bone age advancement occurred in 51.7% of cases. Vitamin D insufficiency or deficiency was detected in 85.6% of patients, and hypocalcemia was present in 85.1%. Overweight/obesity, vitamin D deficiency, and bone age advancement were associated with older age, prolonged corticosteroid therapy, higher androgen levels, and clinical features of treatment imbalance (e.g., Cushingoid appearance, hyperpigmentation). Female sex was significantly associated with higher rates of stunting. **Conclusions**: Growth impairment, nutritional deficiencies, and skeletal maturation disturbances are prevalent among children with CAH in Vietnam. Early identification of risk factors and the implementation of tailored management strategies that address both endocrine and nutritional health are crucial for optimizing long-term outcomes.

## 1. Introduction

Congenital adrenal hyperplasia (CAH) represents a group of autosomal recessive disorders characterized by enzymatic deficiencies in adrenal steroid biosynthesis [1]. Among these, 21-hydroxylase deficiency, caused by mutations in the *CYP21A2* gene, is the most prevalent, accounting for over 90% of cases globally [2]. This enzymatic defect disrupts cortisol and aldosterone production, leading to compensatory stimulation of adrenocorticotropic hormone (ACTH) and excessive adrenal androgen secretion. As a result, affected children may exhibit clinical signs ranging from ambiguous genitalia at birth to early-onset puberty and metabolic disturbances [1].

The global incidence of CAH is estimated to range between 1 in 14,000 and 1 in 18,000 live births [1]. However, recent meta-analyses suggest that incidence rates may be increasing in certain populations, with reports as high as 1 in 9498 births [2,3]. Notably, incidence varies by geographic region and ethnic group, with higher rates observed in the Eastern Mediterranean and Southeast Asian populations [3].

The clinical presentation of CAH in infancy and childhood varies by the severity of the enzyme defect [4]. Classic CAH, particularly the salt-wasting form, often manifests during the neonatal period with vomiting, dehydration, hypotension, and hypoglycemia—hallmarks of adrenal insufficiency. Girls with classic 21-hydroxylase deficiency present with ambiguous genitalia, whereas affected boys exhibit subtle signs such as hyperpigmentation and penile enlargement. Without adequate treatment, prolonged androgen exposure leads to rapid growth, early pubic hair, and advanced bone age, and may trigger centrally mediated precocious puberty [5].

Beyond the endocrine abnormalities, CAH is increasingly recognized for its impact on somatic growth and nutritional health. A 2010 meta-analysis found that adult patients with CAH had a mean height approximately 10 cm below population norms, with a standard deviation score of −1.4 [6]. A 2018 study on children with congenital adrenal hyperplasia reported that 17.6% of patients were obese, and 25.7% exhibited short stature [7]. Obesity is another common finding in pediatric CAH. Approximately one-third of children are overweight or obese, with prevalence increasing markedly by age four [8,9,10].

Micronutrient deficiencies—especially vitamin D—have emerged as prevalent issues in CAH. A 2012 study of 244 patients revealed that 61% had suboptimal vitamin D levels, potentially due to the effects of prolonged corticosteroid therapy [8].

These outcomes are driven by both the disease pathology and its treatment. Excess adrenal androgens can prematurely advance skeletal maturation, compromising final height despite transient increases in growth velocity during early childhood. Studies have shown that affected children, especially those with the classic form, are prone to short stature, obesity, and altered body composition [11,12,13].

Simultaneously, managing the lifelong glucocorticoid therapy required for CAH presents a significant clinical challenge [14]. Clinicians must balance the consequences of undertreatment, such as androgen excess leading to virilization and premature bone age advancement, with the risks of overtreatment from the necessary supraphysiologic steroid doses. Excessive glucocorticoid exposure is well known to suppress linear growth and contributes to obesity, low bone mineral density, and other long-term cardiometabolic complications. These adverse effects tend to become more pronounced in children undergoing treatment for more than five years [10,15,16,17,18]. Furthermore, corticosteroids are known to impair vitamin D metabolism by increasing 24-hydroxylase activity, which accelerates the degradation of 25(OH)D [19].

While international studies have highlighted the long-term health risks in CAH, research in some regions remains limited. In Vietnam, for example, few studies have comprehensively evaluated physical development and nutritional status in children with CAH. A study by Nguyen et al. found that early diagnosis and adequate treatment adherence were critical to optimizing physical development in CAH children [20]. To our knowledge, this is the first study in Vietnam to simultaneously evaluate both growth development and nutritional status in children with CAH. Therefore, gaps remain regarding the long-term implications of glucocorticoid therapy on bone health, nutritional status, and growth outcomes, particularly in resource-limited settings. These challenges are often exacerbated by systemic barriers to care. For instance, a recent review by Eitel and Fechner noted that access to universal newborn screening, widely recognized as the most effective strategy for early diagnosis, remains limited in many low- and middle-income countries [21]. In Vietnam, as of 2019, only an estimated 40% of newborns had access to comprehensive screening programs [22,23]. The potential for delayed diagnosis, combined with constrained access to multidisciplinary care, underscores the urgent need to assess real-world growth and nutritional outcomes in this specific population.

This study aimed to investigate the growth outcomes, nutritional indicators, and associated factors among 201 Vietnamese children with CAH at the Vietnam National Children’s Hospital.

## 2. Materials and Methods

### 2.1. Subjects

This cross-sectional descriptive study was conducted on children diagnosed with congenital adrenal hyperplasia (CAH). Eligible participants were aged 1 to 17 years, in accordance with the World Health Organization’s (WHO) child age classification (WHO, 2006) [24]. All patients included had a confirmed diagnosis of CAH in accordance with the Endocrine Society Clinical Practice Guidelines (2018) [2] (see Supplementary Materials S1) and had been undergoing treatment for at least 12 months.

Exclusion criteria included the presence of other chronic medical conditions or congenital malformations, such as congenital heart disease, epilepsy, or cancer.

The study was approved by the Institutional Review Board of Vietnam National Children’s Hospital (protocol code 2685/BVNTW-HĐĐĐ, approved on 10 October 2024) in accordance with current legislation, the Declaration of Helsinki, and standards of good clinical practice. Informed consent was obtained from all parents or legal guardians of the children included in the study.

The study was conducted from July 2024 to April 2025 at the Center for Endocrinology, Metabolism, Genetics, and Molecular Therapy, Vietnam National Children’s Hospital.

### 2.2. Clinical and Biochemical Assessments

Clinical data were collected through structured interviews, clinical examinations, and retrospective medical record reviews.

In alignment with the 2018 Endocrine Society Clinical Practice Guidelines for the management of congenital adrenal hyperplasia [2], we evaluated clinically relevant variables reflecting both undertreatment and overtreatment. These included signs of inadequate androgen suppression, such as hyperpigmentation, virilization (clitoromegaly in females, penile enlargement in males, hirsutism, acne, and other manifestations of androgen excess), as well as signs of glucocorticoid excess, like Cushingoid features (weight gain, truncal obesity, moon facies, facial plethora) [25] or acanthosis nigricans. Clinical history of adrenal crises was not formally quantified due to the retrospective nature of data collection and the potential for recall bias. Precocious puberty is defined as the appearance of secondary sexual characteristics before age 8 in girls and before age 9 in boys [26]. The diagnosis was based on clinical signs such as breast development in girls or testicular volume ≥ 4 mL in boys, rapid height growth, and bone age advanced by more than two standard deviations [26,27,28,29,30,31]. A baseline LH > 0.3 mIU/mL or stimulated peak LH > 5 mIU/mL confirms CPP, while suppressed LH/FSH with elevated sex steroid levels indicates PPP [32,33,34,35,36,37]. Imaging studies (pelvic/testicular ultrasound, brain MRI) and hormone testing (estradiol, testosterone, 17-OHP) were used to confirm the diagnosis and assess underlying etiologies [34,36,38,39,40,41,42,43].

Anthropometric assessments included weight, length/height, and body mass index (BMI), evaluated in accordance with the WHO Growth Standards (WHO 2006 for children under 5 years [24] and WHO 2007 for those aged 5 years and older [44]):

BMI: For children < 5 years—severe thinness (<−3 SD), thinness (−3 to <−2 SD), normal (−2 to +2 SD), overweight (>+2 to +3 SD), obesity (>+3 SD); for children ≥ 5 years—severe thinness (<−3 SD), thinness (−3 to <−2 SD), normal (−2 to +1 SD), overweight (>+1 to +2 SD), obesity (>+2 SD).

Height-for-age z-score (HAZ): normal (HAZ ≥ −2 SD), stunted (HAZ < −2 SD), and severely stunted (HAZ < −3 SD).

Weight was measured using a digital scale, and height or length was assessed using a stadiometer or recumbent length board, depending on the child’s age and ability to stand.

Bone age was determined from standard radiographs of the left hand and wrist performed at the Department of Diagnostic Imaging using the Carestream DRX1-System (Carestream Health, Washington, DC, USA). All images were interpreted by trained radiologists employing the Greulich and Pyle atlas. BA-CA Classification: Categorization of skeletal maturation based on the comparison between bone age (BA) and chronological age (CA), where BA > CA indicates advanced bone age, BA = CA indicates bone age matches chronological age, and BA < CA indicates delayed bone age.

Biochemical analyses included measurements of total serum calcium, ionized calcium, and serum phosphate, performed using colorimetric absorption methods on a Beckman Coulter AU5800 analyzer (Beckman Coulter, Tokyo, Japan). Serum 25-hydroxyvitamin D [25(OH)D] levels were determined by electrochemiluminescence immunoassay on the same analyzer. Serum 17-hydroxyprogesterone was quantified using a semi-automated ELISA on a Biotek system (Biotek, Winooski, VT, USA), and testosterone levels were measured via electrochemiluminescence immunoassay on a Cobas E601 analyzer (Roche Diagnostics, Tokyo, Japan). All laboratory analyses were conducted at the Biochemistry Department. The reference ranges for each parameter were applied accordingly:

- 25-hydroxyvitamin D [25(OH)D]: deficiency: <50 nmol/L; insufficiency: 50–72.5 nmol/L [45].
- Total serum calcium, 2.2–2.7 mmol/L; ionized calcium, 1.12–1.23 mmol/L. Serum phosphate levels were interpreted using age-specific reference ranges as follows: 1.25–2.10 mmol/L for children aged 1–3 years, 1.20–1.80 mmol/L for ages 4–11 years, 0.95–1.75 mmol/L for ages 12–15 years, and 0.90–1.50 mmol/L for ages 16–19 years [46].
- 17-hydroxyprogesterone (17-OHP) and testosterone levels were interpreted according to age- and sex-specific reference standards.

The following treatment-related variables were collected: type of glucocorticoid, current daily dose, dosing frequency, and duration of therapy. All glucocorticoid doses were standardized by converting to hydrocortisone equivalents, using the following conversion ratios: 1 mg prednisolone = 5 mg hydrocortisone, and 1 mg dexamethasone = 80 mg hydrocortisone [19,47].

### 2.3. Data Analysis

All data were entered and processed using SPSS version 20.0 (IBM Corp., Armonk, NY, USA). The distribution of continuous variables was assessed using the Kolmogorov–Smirnov test, which is appropriate for sample sizes greater than 50. Continuous variables were presented as mean ± standard deviation (SD) if normally distributed or as median (minimum–maximum) if non-normally distributed. Categorical variables were expressed as frequencies and percentages.

Comparisons between groups were conducted using the following statistical tests: Independent *t*-test and ANOVA for normally distributed continuous variables; Mann–Whitney U test and Kruskal–Wallis test for non-parametric variables; chi-squared test and Fisher’s exact test for categorical variables depending on sample size. A *p*-value of <0.05 was considered statistically significant. In addition to *p*-values, effect sizes or measures of magnitude (e.g., mean differences, odds ratios, or relevant coefficients) were reported where applicable. Quality control measures included predefined inclusion/exclusion criteria, the use of standardized case report forms, and double data entry for validation. For variables with less than 20% missing data—including 17-hydroxyprogesterone (17OHP), ionized calcium, and the difference between bone age and chronological age (BA-CA)—missing values were imputed using the median of the respective variable. This approach was selected to preserve the distributional characteristics of non-normally distributed variables and to minimize potential bias. No variable exceeded the 20% missing data threshold that would require exclusion from analysis.

## 3. Results

### 3.1. Description of the Study Sample

**Figure 1:**
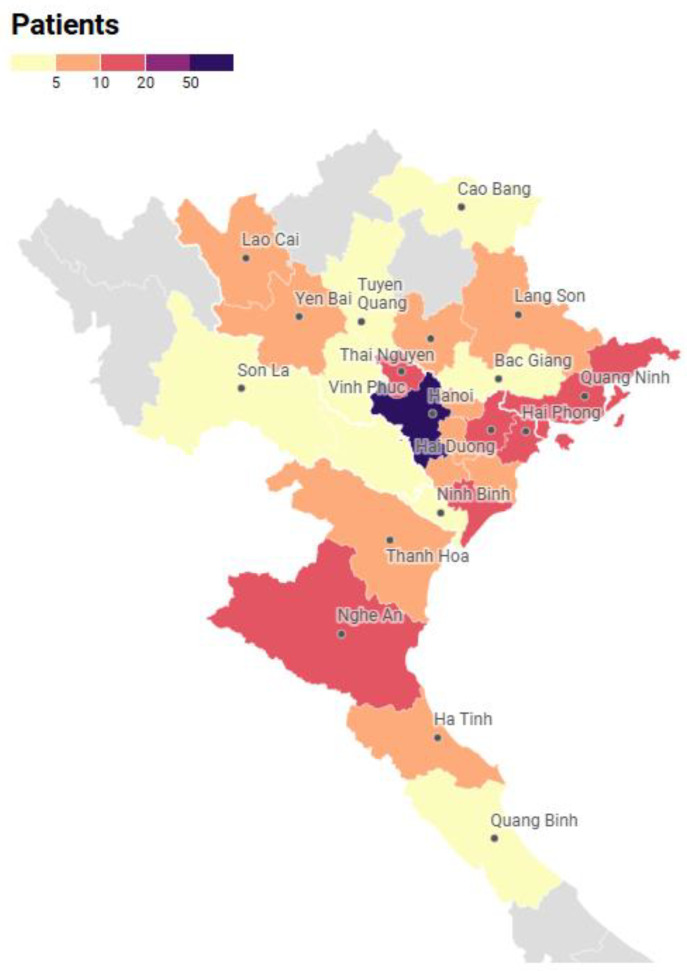
Geographic distribution of the CAH patient population (*n* = 201).

A total of 201 children diagnosed with CAH were included in the study. The median age at valuation was 9.8 years, ranging from 1.1 to 16.5 years. Participants originated from 25 provinces across the northern and central regions of Vietnam (Figure 1). The geographic distribution analysis indicated regional variations in the number of reported CAH cases, with higher concentrations observed in certain provinces.

### 3.2. Growth Assessment and Nutritional Status

**Figure 2:**
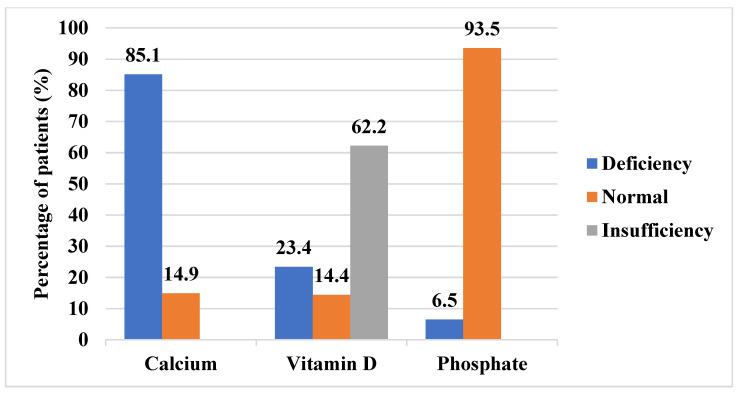
Distribution of calcium, vitamin D, and phosphate status.

Anthropometric measurements revealed that the median height standard deviation score (SDS) was −0.7 (range: −4.0 to 4.3), and the median body mass index (BMI) SDS was 1.4 (range: −2.7 to 5.2). The median difference between bone age and chronological age (BA-CA) was 1.4 years (range: −8.3 to 8.6), indicating a general trend toward advanced skeletal maturation. Among the 201 patients, 51.7% had an advanced bone age (BA > CA), 38.3% had an age-appropriate bone age (BA = CA), and 10.0% had a delayed bone age (BA < CA). In terms of BMI, 1.5% were classified as thin, 45.3% had normal weight, 24.4% were overweight, and 28.9% were obese. Regarding height-for-age, 4.5% had severe stunting, 11.9% had moderate stunting, and 83.6% had normal stature. Vitamin D insufficiency or deficiency was observed in 85.6% of the sample, while 85.1% exhibited calcium deficiency. Among the 138 patients with available phosphate data, 6.5% were found to have phosphate deficiency (Figure 2). The baseline sociodemographic, clinical, biochemical, and anthropometric characteristics of the study population are presented in Table 1.

### 3.3. Sociodemographic Factors

#### 3.3.1. Age

Age was significantly associated with several key study outcomes. Children presenting with advanced bone age (BA > CA), higher BMI classifications (overweight/obesity), and vitamin D deficiency were significantly older than their respective comparison groups (*p* < 0.001 for each). A significant association was also observed with hypophosphatemia (*p* = 0.039). Conversely, no significant age differences were found for height-for-age classifications (*p* = 0.183) or the presence of hypocalcemia (*p* = 0.24). The distribution of age across nutritional and biochemical outcomes is summarized in Table 2.

#### 3.3.2. Gender Differences

Gender-specific differences were observed in growth parameters among children with CAH. Both hypocalcemia and hypophosphatemia were highly prevalent in males and females, with no statistically significant differences between sexes (*p* = 0.168 and *p* = 0.316, respectively). Similarly, vitamin D status did not differ significantly by gender (*p* = 0.875).

Regarding nutritional status, obesity was more frequently observed in males than in females (35.1% vs. 23.1%), although this difference was not statistically significant (*p* = 0.151), indicating a trend toward significance (*p* = 0.057). In contrast, stunting was significantly more common among females, with 23.1% exhibiting moderate to severe stunting compared to 9.3% of males (*p* = 0.003).

In terms of bone age, delayed maturation was more frequent in females (13.5% vs. 6.2%), though the difference did not reach statistical significance (*p* = 0.163).

#### 3.3.3. Residential Area

No significant differences were observed in the prevalence of hypocalcemia and hypophosphatemia between urban and rural children (*p* = 0.073 and *p* = 0.186, respectively). Vitamin D deficiency was more prevalent among urban children (28.6%) compared to their rural counterparts (16.9%), although the difference was not statistically significant (*p* = 0.104).

Similarly, the combined prevalence of overweight and obesity was higher in urban areas (59.8% combined) than in rural areas (49.4%), but the difference did not reach statistical significance (*p* = 0.093). No significant differences were found between urban and rural groups in height-for-age classification (*p* = 0.218) or patterns of bone age advancement (*p* = 0.956).

### 3.4. Disease-Related Factors

#### 3.4.1. Clinical Phenotype

No significant differences were observed between salt-wasting and simple virilizing forms in the prevalence of hypocalcemia (*p* = 0.298), vitamin D status (*p* = 0.337), BMI classification (*p* = 0.796), or height-for-age classification (*p* = 0.203). In contrast, hypophosphatemia was significantly more prevalent in children with the simple virilizing form compared to those with the salt-wasting form (18.5% vs. 3.6%, *p* = 0.014). Moreover, bone age advancement was substantially more frequent in the simple virilizing group, with 82.9% of individuals showing advanced bone age, compared to 43.8% in the salt-wasting group (*p* < 0.001).

#### 3.4.2. Clinical Disease Control Indicators

Obesity was significantly more prevalent among children with Cushingoid features (41.2% vs. 27.7%, *p* = 0.001, Cramer’s V = 0.262) and among those with acanthosis nigricans (80.0% vs. 24.7%, *p* < 0.001, Cramer’s V = 0.334) compared to their respective counterparts. Children with hyperpigmentation also demonstrated higher rates of obesity (40.0% vs. 25.2%, *p* = 0.045, Cramer’s V = 0.201), as well as more frequent bone age advancement (70.0% vs. 45.7%, *p* = 0.005, Cramer’s V = 0.227). Similarly, bone age advancement was more prevalent in children with virilization (77.4% vs. 47.1%, *p* = 0.004, Cramer’s V = 0.231) and those with central precocious puberty (80.9% vs. 42.9%, *p* < 0.001, Cramer’s V = 0.324).

The prevalence of hypocalcemia was significantly higher among children with hyperpigmentation compared to those without (96.0% vs. 81.5%, respectively; *p* = 0.012, OR = 5.46, 95% CI: 1.25–23.83). Similarly, children with central precocious puberty (CPP) had a significantly higher prevalence of hypocalcemia than those without CPP (95.7% vs. 81.8%; *p* = 0.019, OR = 5.00, 95% CI: 1.145–21.84). Hypophosphatemia was also significantly more common in the CPP group compared to the non-CPP group (14.7% vs. 3.8%; *p* = 0.041, OR = 4.31, 95% CI: 1.086–17.103). No other significant associations were found between the clinical features and the assessed nutritional or growth outcomes (Table 3).

#### 3.4.3. Biochemical Markers

Median 17-hydroxyprogesterone (17-OHP) levels were higher in children with hypocalcemia (52.4 nmol/L) than in those without hypocalcemia (39.7 nmol/L), although this difference approached but did not reach statistical significance (*p* = 0.137). In contrast, median testosterone concentrations were significantly elevated in the hypocalcemia group (0.24 nmol/L) compared to the non-hypocalcemia group (0.09 nmol/L, *p* = < 0.001).

Regarding hypophosphatemia, children with the condition had slightly lower median 17-OHP levels compared to those without (47.8 vs. 55.1 nmol/L); however, this difference was not statistically significant (*p* = 0.103). Likewise, testosterone levels did not differ significantly between the groups (*p* = 0.543). Testosterone levels also varied significantly by vitamin D status (*p* = 0.027), with lower median concentrations observed in children with normal vitamin D levels (0.09 nmol/L) compared to those with deficiency or insufficiency (both 0.24 nmol/L). In contrast, median 17-OHP levels did not differ significantly across vitamin D categories: 55.8 nmol/L in the deficient group, 52.35 nmol/L in the insufficiency group, and 31.3 nmol/L in the normal group (*p* = 0.317).

Regarding growth parameters, 17-OHP levels differed significantly across BMI categories (*p* = 0.014), with the highest median concentration observed in children with obesity (107.5 nmol/L). In contrast, testosterone levels did not significantly vary by BMI classification (*p* = 0.083).

In relation to height-for-age, children with severe stunting showed a trend toward lower testosterone levels (median 0.09 nmol/L) compared to those with moderate stunting or normal status (both 0.24 nmol/L), although the difference did not reach statistical significance (*p* = 0.053). No significant differences were observed in 17-OHP levels across height-for-age groups (*p* = 0.131).

Bone age advancement was significantly associated with hormonal levels. Children with an advanced bone age (BA > CA) had the highest median testosterone levels (0.78 nmol/L, *p* < 0.001) and 17-OHP levels (69.6 nmol/L, *p* = 0.005) compared to those whose BA was equal to or less than their chronological age.

### 3.5. Treatment-Related Factors

Children with hypocalcemia had a significantly longer treatment duration compared to those without (median, 8.9 vs. 2.8 years; *p* < 0.001; r = 0.447), indicating a moderate to large effect size. In contrast, there was no significant difference in treatment duration for children with or without hypophosphatemia (*p* = 0.942; r = 0.006). Treatment duration also varied by vitamin D status (*p* = 0.007; η^2^ = 0.04). Children with vitamin D deficiency had the longest treatment (median 9.7 years), followed by those with insufficiency (8.5 years) and normal levels (5.3 years). This suggests a small but relevant effect.

Regarding BMI, treatment was longer in obese (10.1 years) and overweight (9.1 years) children compared to those with a normal BMI (5.8 years) or thinness (2.5 years; *p* < 0.001; η^2^ = 0.08), reflecting a moderate effect. No significant difference was found by height-for-age classification (*p* = 0.108; η^2^ = 0.01). For bone age, children with advanced bone age (BA > CA) had a longer treatment duration (median, 10.2 years) compared to those with bone age equal to chronological age (5.8 years) or delayed bone age (4.6 years; *p* < 0.001; η^2^ = 0.10). This indicates a moderate effect. These associations between glucocorticoid treatment duration and growth or nutritional parameters are summarized in Table 4.

### 3.6. Associations Between Subclinical Nutritional and Growth Outcomes

In the BA-CA classification groups, hypocalcemia was significantly more prevalent among children with advanced bone age (BA > CA) at 96.2%, compared to those with age-appropriate (72.7%) or delayed bone age (75.0%) (*p* < 0.001). Vitamin D deficiency was also more frequent in the advanced bone age group (26.9%) than in those with bone age equal to (19.5%) or delayed relative to chronological age (20.0%). This trend was statistically significant, as confirmed by linear-by-linear association (*p* = 0.036). In contrast, no significant association was found between bone age classification and the prevalence of hypophosphatemia (*p* = 0.457).

When stratified by height-for-age categories, no significant differences were observed in the prevalence of hypocalcemia, hypophosphatemia, or vitamin D status. However, analysis by BMI classification revealed a significantly higher prevalence of hypocalcemia among overweight (93.9%) and obese (91.4%) children compared to those with normal BMI (76.9%) or thinness (66.7%) (*p* = 0.016). The distribution of vitamin D status across BMI categories showed a trend toward significance (*p* = 0.063) and a significant linear association (*p* = 0.012); vitamin D deficiency was more common in overweight (29.3%) and obese children (30.6%) than in those with normal weight (16.5%). No significant association was found between BMI classification and hypophosphatemia (*p* = 0.76). Children with hypocalcemia had a significantly different distribution of vitamin D status compared to those without hypocalcemia (*p* = 0.016), characterized by a higher prevalence of vitamin D deficiency (25.7% vs. 10%) and a lower proportion of normal vitamin D status (11.7% vs. 30%).

## 4. Discussion

This study provides a detailed clinical and biochemical profile of 201 Vietnamese children with congenital adrenal hyperplasia (CAH), highlighting growth impairment, skeletal maturation disturbances, nutritional deficits, and treatment-related effects. Our findings are generally consistent with international literature while offering novel insights from a Southeast Asian context.

### 4.1. Growth and Nutritional Outcome

In our study, the median height standard deviation score (SDS) was −0.7 (range: −4.0 to 4.3), and 16.4% of participants were classified as stunted (11.9% moderate, 4.5% severe). This finding aligns with previous studies, where Muthusamy et al. reported a pooled mean height standard deviation (SDS) of −1.03 in a meta-analysis [6] and Gidlöf et al. found a final height SDS of −0.78 in an extensive European registry [48]. Moreover, studies have indicated that during adrenarche and early puberty, children with CAH may initially appear taller than their peers due to accelerated skeletal maturation, but ultimately achieve a lower final adult height if skeletal advancement persists [49]. In our study, although early bone age advancement was common (51.7%), this did not necessarily translate into preserved final height, emphasizing the need for careful longitudinal growth monitoring. A key finding in our study was the significantly higher rate of stunting among females (23.1%) compared to males (9.3%). This gender dichotomy is strongly supported by previous longitudinal research. A study by Patel et al. specifically evaluating growth trajectories in children with 21-hydroxylase deficiency found that growth curves for height, weight, and BMI differed significantly by gender [50]. They reported that females, in contrast to males, showed a pattern of disproportionately greater adiposity and increasing BMI but had shorter stature during adolescence. In their cohort, females with salt-wasting CAH initially experienced less growth retardation than males but ultimately developed a greater discrepancy between their weight and height scores. Meanwhile, females with non-salt-wasting CAH showed only a slight gain in height SDS, while their weight and BMI SDS progressively increased throughout childhood.

In an attempt to explain this, Patel et al. noted that both sexes in their study received comparable doses of corticosteroids, indicating that the difference in height outcome was not simply related to steroid treatment. The authors speculated that other factors, such as prenatal programming due to androgen excess in utero, may contribute to these different postnatal growth patterns, with a more pronounced adverse effect on height in females.

Overweight and obesity were highly prevalent in our cohort, with 24.4% classified as overweight and 28.9% as obese, totaling 53.3%, similar to rates reported by Abdel Meguid et al., who observed a 60% prevalence among Egyptian CAH children [17]. This is notably higher than the 16.5% prevalence found by Völkl et al. in a German cohort [51], possibly reflecting differences in genetic background, treatment regimens, or lifestyle factors. Importantly, we found that obesity was more frequent in males (35.1%) than in females (23.1%) and increased significantly with age (median age of obese children: 10.5 years, *p* < 0.001), which supports the findings of Gidlöf et al. [48]. The pathophysiological mechanisms underlying obesity in CAH are multifactorial, including supraphysiological glucocorticoid exposure leading to central adiposity [52], altered insulin sensitivity [53], and the anabolic effects of chronic hyperandrogenism [54].

However, those findings should be interpreted within the context of the current nutritional landscape in Vietnam. According to the 2019–2020 General Nutrition Survey, the nationwide prevalence of stunting in children aged 5 to 19 years was 14.8%, a figure comparable to the rate in our cohort [55]. This suggests that stunting may be a persistent public health challenge in the region, potentially exacerbated by the effects of CAH and its treatment. More strikingly, the 53.3% prevalence of overweight and obesity in our study is substantially higher than the national average of 19.0% reported for school-aged children in the same survey. While rising obesity rates are a general concern in Vietnam, particularly in urban areas, the markedly higher prevalence in our patients strongly suggests that disease-specific factors, such as supraphysiologic glucocorticoid dosing and metabolic alterations inherent to CAH, are major contributors to this adverse outcome.

Skeletal maturation disturbances were also prominent. In our study, 51.7% of patients had an advanced bone age, which was more common in the simple virilizing form (82.9%) compared to the salt-wasting form (43.8%, *p* < 0.001), consistent with the findings by Bomberg et al. [56]. Furthermore, we found that children with advanced bone age exhibited higher median testosterone and 17-OHP levels. This observation is supported by Finkielstain et al., who reported a significant correlation between bone age advancement and elevated concentrations of androstenedione and testosterone [9]. Such findings reflect the pathophysiological mechanism of chronic ACTH stimulation, leading to excess adrenal androgen production and promoting premature epiphyseal fusion, thereby compromising linear growth potential.

Vitamin D insufficiency or deficiency, as well as hypocalcemia, were prevalent, affecting 85.6% and 85.1% of our patients, respectively. This finding mirrors the high prevalence of vitamin D insufficiency (74.9%) reported by Demirel et al. in children with CAH [57] and aligns with the findings by Finkielstain et al., who noted that 19% of classic CAH patients were vitamin D deficient and 42% were insufficient [8]. However, to provide a direct comparative context for our findings, a key study by Laillou et al. on healthy Vietnamese children reported that 58% had 25(OH)D levels below 50 nmol/L [58]. This figure comprised 21% of children classified as deficient (<30 nmol/L) and 37% classified as insufficient (30–49.9 nmol/L). When more liberal diagnostic thresholds were applied (25(OH)D <75 nmol/L), approximately 90% of healthy Vietnamese children were classified as having hypovitaminosis D. Furthermore, the same study found that 97% of children had mild hypocalcemia. These deficiencies in the general pediatric population were linked to extremely low dietary intake, with children consuming only about 1% of the recommended nutrient intake for vitamin D and less than 43% for calcium. Given this high baseline prevalence of vitamin D and calcium deficiencies in the general Vietnamese pediatric population, it is likely that the rates observed in our CAH cohort are compounded by these widespread environmental and dietary factors rather than being solely a consequence of CAH or its treatment.

Additionally, vitamin D deficiency increased with age and BMI, being more prevalent in older children (10.3 vs. 5.6 years, *p* = 0.001) and those with obesity (29.3% vs. 16.5%, *p* = 0.012), consistent with patterns in steroid-treated pediatric populations [38].

### 4.2. Related Factors

Prolonged corticosteroid therapy was found to have a significant impact on growth and metabolic outcomes in children with CAH. In our study, children with obesity had a longer median treatment duration (10.1 years) compared to those with a normal BMI (5.8 years, *p* < 0.001). Additionally, children with hypocalcemia received corticosteroids for a median of 8.9 years, compared to 2.8 years for those without (*p* < 0.001). Additionally, corticosteroid duration varied significantly with vitamin D status (*p* = 0.007), being longest in children with deficiency (9.7 years) and shortest in those with normal levels (5.3 years). These findings are consistent with previous studies. Abdel Meguid et al. reported that children who had been treated with corticosteroids for more than five years exhibited a markedly higher prevalence of overweight and obesity, reaching 60% [17]. Similarly, Finkielstain et al. observed that 35% of children with CAH were obese, with a higher proportion receiving long-acting glucocorticoid formulations compared to non-obese peers [8]. In addition to its impact on body composition, corticosteroid therapy adversely affected linear growth. Stikkelbroeck et al. demonstrated that higher cumulative glucocorticoid doses were significantly associated with reduced height-for-age z-scores (HAZ) during critical periods of growth, specifically between 6 and 12 months and between 8 and 14 years of age [16]. These findings support the well-established notion that supraphysiological glucocorticoid exposure suppresses chondrocyte proliferation and disrupts the growth plate, ultimately impairing linear growth potential. Moreover, corticosteroid use was associated with disturbances in vitamin D metabolism. Demirel et al. reported that children receiving supraphysiological steroid doses (>15 mg/m^2^/day) exhibited lower serum 25-hydroxyvitamin D levels compared to those on physiological doses, although the difference did not reach statistical significance [57]. These findings suggest that glucocorticoid therapy may contribute to impaired vitamin D status through indirect mechanisms, thereby exacerbating the risk of bone mineral disturbances and hypocalcemia observed in patients with CAH.

Higher BMI categories were significantly associated with a greater prevalence of vitamin D deficiency in this study. Among obese children, the deficiency rate was 29.3%, compared to 16.5% in those with normal BMI, with a significant linear trend across BMI classifications (*p* = 0.012). The proportion of vitamin D sufficiency was also lower in obese participants (10.3%) than in their normal-weight peers (22%), reinforcing the link between adiposity and impaired vitamin D status. These findings are consistent with those of Demirel et al., who reported that children with higher BMI tended to have lower 25-hydroxyvitamin D concentrations. However, the association did not reach statistical significance [57]. This relationship is biologically plausible, given that vitamin D, as a fat-soluble vitamin, may be sequestered in adipose tissue, thereby reducing its circulating bioavailability [59]. Consequently, children with higher BMI may be at greater risk for hypovitaminosis D, compounding the nutritional and skeletal challenges already faced by CAH patients.

Among our patients, elevated 17-hydroxyprogesterone (17-OHP) and testosterone levels were significantly associated with adverse growth and skeletal outcomes. Median 17-OHP was higher in children with hypocalcemia (52 vs. 39.7 nmol/L, *p* = 0.317) and obesity (107.5 nmol/L in obese children, *p* = 0.014). Testosterone levels were significantly higher in individuals with hypocalcemia (*p* < 0.001), vitamin D deficiency (*p* = 0.027), and advanced bone age (*p* < 0.001). These findings reflect the impact of poor biochemical control on mineral metabolism, body composition, and skeletal maturation. Children with advanced bone age exhibited the highest median 17-OHP and testosterone levels, reinforcing the contribution of androgen excess to accelerated skeletal development. This is consistent with prior studies, where elevated 17-OHP concentrations (>30 nmol/L) during adrenarche were associated with lower estimated final height [49], and testosterone elevations were significantly linked to bone age advancement [8]. Although children with central precocious puberty (CPP) demonstrated higher testosterone concentrations, this was not associated with significant height impairment at the time of evaluation in our cohort.

In our cohort, several clinical features demonstrated significant associations with adverse growth and nutritional outcomes. Cushingoid appearance and acanthosis nigricans were strongly associated with higher BMI classifications, reflecting the impact of chronic glucocorticoid excess and consequent insulin resistance. Indicators of poor biochemical control, particularly hyperpigmentation, were also linked to multiple systemic disturbances. Hyperpigmentation was associated with a higher prevalence of obesity, more frequent bone age advancement, and a significantly increased prevalence of hypocalcemia, suggesting that chronic ACTH elevation may exacerbate these conditions. Among undertreatment indicators, virilization correlated substantially with advanced bone age classification, consistent with the effect of androgen excess on premature skeletal maturation. The presence of CPP was significantly associated not only with profound bone age advancement but also with a higher prevalence of both hypocalcemia and hypophosphatemia. These results underscore that inadequate androgen suppression, particularly to the point of inducing CPP, has profound effects on both skeletal and mineral metabolism in children with CAH.

Our findings complement earlier Vietnamese studies. Nguyen et al. [20] conducted a study of 124 pediatric CAH patients and found that early diagnosis, sufficient steroid dosing, and treatment adherence were key to achieving good treatment outcomes. However, their study did not include biochemical or nutritional indicators [18].

The limitations of this study include its cross-sectional design, which precludes causal inference, and the absence of bone mineral density assessments, genotypic data, and lipid or insulin profiling. In our study, we did not observe statistically significant differences in calcium, phosphorus, or vitamin D levels between the salt-wasting and simple virilizing forms of CAH. However, genotypic data were not available for this cohort, and thus, we could not evaluate whether biochemical differences exist across specific 21-hydroxylase mutation types. Given the established correlation between genotype and phenotype severity, future studies should incorporate molecular characterization to determine whether certain mutations are associated with distinct patterns of micronutrient abnormalities or bone mineral status. Additionally, pubertal development was not assessed using Tanner staging, limiting the ability to precisely interpret hormone levels and skeletal maturation in relation to pubertal status. Although we evaluated biochemical markers of nutritional status, dietary intake data, such as food frequency questionnaires or detailed nutrient intake records, were not collected, which limited our ability to directly correlate micronutrient deficiencies with dietary patterns. Phosphate measurements were available in a subset of patients, which limited the statistical power for analyses involving phosphate deficiency. While this did not affect the primary study objectives, the results related to phosphate status should be interpreted with caution. Another significant limitation of this study is the lack of established local pediatric biochemical reference intervals for the Vietnamese population. To maintain consistency, we adopted age-stratified reference ranges from a major pediatric textbook (Nelson Textbook of Pediatrics) for our definitions of deficiency [46]. We fully acknowledge that these international ranges, likely based on Western populations, may not perfectly reflect the biochemical norms for Vietnamese children. This limitation introduces the potential for misclassification, either overestimating or underestimating the true prevalence of nutritional deficiencies in our cohort. Future research should aim to fill this gap by conducting population-based studies to define national biochemical reference values. Additionally, standardized pubertal staging, comprehensive dietary assessments, and behavioral evaluations should be integrated into future studies to elucidate better the complex nutritional and metabolic risk profiles of children with CAH. Nonetheless, the study’s large sample size, integrated clinical and hormonal data, and inclusion of both urban and rural populations provide valuable insights into CAH management in resource-constrained settings.

## 5. Conclusions

In a Vietnamese pediatric cohort with congenital adrenal hyperplasia, we identified a high prevalence of overweight/obesity, vitamin D deficiency, hypocalcemia, and advanced bone age, despite standard treatment. Significant associations were found between adverse outcomes and clinical indicators of treatment imbalance, androgen excess, prolonged corticosteroid exposure, and residential area. These findings reflect not only disease-specific challenges but also broader nutritional and healthcare disparities within the Vietnamese context. Our results underscore the urgent need for individualized, comprehensive management strategies that address both endocrine control and nutritional status to improve long-term health outcomes in this population.

## Figures and Tables

**Table 1:** Clinical characteristics of pediatric patients with CAH.

		Salt-Wasting	Simple Virilizing	Total
Sociodemographic	n	160	41	201
	Female, n (%)	50.6 (81)	56.1 (23)	104 (51.7)
	Age (year)	8.7 ± 4.3	9.8 ± 3.6	9.8 (1.1–16.5)
	Urban area, n (%)	92 (57.5)	20 (48.8)	112 (55.7)
Clinical characteristics	Height SDS	−0.9 (−4.0–2.9)	0.4 (−2.6–4.3)	−0.7 (−4.0–4.3)
	Normal, n (%)	130 (81.2)	38 (92.7)	168 (83.6)
	Moderate stunting, n (%)	21 (13.1)	3 (7.3)	24 (11.9)
	Severe stunting, n (%)	9 (5.6)	0 (0)	9 (4.5)
	BMI SDS	1.4 (−2.7–5.2)	1.2 (−1.0–3.4)	1.4 (−2.7–5.2)
	Thinness, n (%)	3 (1.9)	0 (0)	3 (1.5)
	Normal, n (%)	71 (44.4)	20 (48.8)	91 (45.3)
	Overweight, n (%)	41 (25.6)	8 (19.5)	49 (24.4)
	Obesity, n (%)	45 (28.1)	13 (31.7)	58 (28.9)
	Cushingoid appearance, n (%)	13 (8.1)	4 (9.8)	17 (8.5)
	Hyperpigmentation, n (%)	44 (27.5)	6 (14.6)	50 (24.9)
	Virilization, n (%)	23 (14.4)	8 (19.5)	31 (15.4)
	Central precocious puberty, n (%)	27 (16.9)	20 (48.8)	47 (23.4)
Biochemical and radiological markers	17OHP (nmol/L)	52.4 (0–1182)	57.1 (2.0–925)	52.4 (0–1182)
	Testosterone (nmol/L)	0.24 (0.1–26.3)	0.78 (0.1–29.8)	0.24 (0.1–29.8)
	Bone age (BA) − Chronological age (CA) (year) ^1^	0.9 (−8.3–6.5)	2.5 (−1.4–8.6)	1.4 (−8.3–8.6)
	Advanced bone age (BA > CA), n (%)	70 (43.8)	34 (82.9)	104 (51.7)
	Age-appropriate bone age (BA = CA), n (%)	71 (44.4)	6 (14.6)	77 (38.3)
	Delayed bone age (BA < CA), n (%)	19 (11.9)	1 (2.4)	20 (10)
Treatment-related factors	Age at diagnosis and treatment (year) ^2^	0 (0–3)	3 (0–9)	0 (0–9)
	Hydrocortisone (mg/m^2^/day)	16.3 ± 4.4	18.7 ± 3.7	16.8 ± 4.4
	Duration of glucocorticoid therapy (year)	8.6 ± 4.3	6.7 ± 3.6	8.2 ± 4.2

^1^ The difference between a patient’s bone age and their chronological age; ^2^ Age at diagnosis and initiation of treatment occurred concurrently and is presented as a single variable.

**Table 2:** Median age and statistical comparisons across nutritional and biochemical outcomes in children with CAH.

Independent Factor	Variable Group	Subgroup	n	Median Age (Years)	*p*-Value
Age at evaluation	Hypocalcemia	Yes	171	0	0.24
		No	30	0	
	Hypophosphatemia	Yes	9	1.5	0.039 *
		No	129	0	
	Vitamin D Status	Deficiency	47	10.3	0.001 *
		Insufficiency	125	10.1	
		Sufficiency	29	5.6	
	BMI Classification	Thinness	3	2.5	<0.001 *
		Normal	91	7.5	
		Overweight	49	10.8	
		Obesity	58	10.5	
	Height-for-Age	Severe stunting	9	4.8	0.183
		Moderate stunting	24	12.7	
		Normal	168	9.7	
	BA-CA Classification	BA > CA	104	10.9	<0.001 *
		BA = CA	77	5.9	
		BA < CA	20	4.6	

* Variable with a significant effect.

**Table 3:** Associations between clinical features and nutritional or growth outcomes in children with CAH.

	Cushingoid Appearance		Acanthosis Nigricans		Hyperpigmentation		Virilization		Central Precocious Puberty	
	*p*-Value	OR (95% CI)	*p*-Value	OR (95% CI)	*p*-Value	OR (95% CI)	*p*-Value	OR (95% CI)	*p*-Value	OR (95% CI)
Hypocalcemia	1 (FET)	0.803 (0.22–2.98)	0.704 (FET)	2.59 (0.33–20.43)	0.012 (Χ^2^) *	5.46 (1.25–23.83)	0.054 (FET)	6.17 (0.81–47.07)	0.019 (Χ^2^) *	5 (1.145–21.84)
Hypophosphatemia	1 (FET)	NA	1 (FET)	NA	0.121 (FET)	3.357 (0.85–13.22)	0.626 (FET)	1.65 (0.319–8.572)	0.041 (FET) *	4.31 (1.086–17.103)
	*p*-value	Cramer’s V	*p*-value	Cramer’s V	*p*-value	Cramer’s V	*p*-value	Cramer’s V	*p*-value	Cramer’s V
Vitamin D Status	0.933 (FET)	0.053	0.741 (FET)	0.073	0.131 (Χ^2^)	0.142	0.128 (FET)	0.139	0.142 (Χ^2^)	0.139
BMI Classification	0.001 (FET) *	0.262	< 0.001 (FET) *	0.334	0.045 (FET) *	0.201	0.779 (FET)	0.083	0.392 (FET)	0.132
Height-for-age	0.64 (FET)	0.059	0.852 (FET)	0.08	0.74 (FET)	0.069	0.206 (FET)	0.129	0.168 (FET)	0.138
BA-CA Classification	0.43 (FET)	0.088	0.205 (FET)	0.135	0.005 (FET) *	0.227	0.004 (FET) *	0.231	<0.001 (FET) *	0.324

* Variable with a significant effect. Values in the table represent *p*-values. Statistical tests used include the chi-squared test (χ^2^) and Fisher’s exact test (FET), depending on expected cell counts. NA indicates comparisons where odds ratios could not be calculated due to zero counts in one or more cells.

**Table 4:** Associations between glucocorticoid treatment duration and nutritional or growth outcomes in children with congenital adrenal hyperplasia (CAH).

	Outcome Variable	*p*-Value	Effect Size
Glucocorticoid treatment duration	Hypocalcemia	<0.001 *	r = 0.447
	Hypophosphatemia	0.942	r = 0.006
	Vitamin D status	0.007 *	η^2^ = 0.04
	BMI classification	<0.001 *	η^2^ = 0.08
	Height-for-age classification	0.108	η^2^ = 0.01
	BA-CA classification	<0.001 *	η^2^ = 0.10

* Variable with a significant effect.

## Data Availability

The original contributions presented in this study are included in the article. Further inquiries can be directed to the corresponding authors.

## Supplementary Materials

The following supporting information can be downloaded at: https://www.mdpi.com/article/10.3390/diagnostics15121534/s1.

## Abbreviations

The following abbreviations are used in this manuscript:

ACTH	Adrenocorticotropic hormone
BA	Bone age
BMI	Body mass index
CA	Chronological age
CAH	Congenital adrenal hyperplasia
CPP	Central precocious puberty
ELISA	Enzyme-linked immunosorbent assay
HAZ	Height-for-age
SD	Standard deviation
SDS	Standard deviation score
SV	Simple virilizing
SW	Salt-wasting
WHO	World Health Organization
17OHP	17-hydroxyprogesterone
25(OH)D	25-hydroxyvitamin D
