# Supplementary material for: Growth Assessment and Nutritional Status in Children with Congenital Adrenal Hyperplasia—A Cross-Sectional Study from a Vietnamese Tertiary Pediatric Center
Source: Diagnostics (Basel). 2025 Jun 16;15(12):1534. doi: 10.3390/diagnostics15121534 (PMC12192552; doi:10.3390/diagnostics15121534)
Supplement: Supplementary file 1 [file diagnostics-15-01534-s001.zip › diagnostics-3663081-supplementary.pdf]

Supplementary File S1. Diagnostic Evaluation and Screening for Congenital Adrenal Hyperplasia (CAH) due to 21-hydroxylase deficiency according to the Endocrine Society 2018 Guidelines [1]

1. Newborn Screening for CAH

- **Recommendation 1.1**: All newborn screening programs should include CAH due to 21-hydroxylase deficiency (21OHD).

2. Initial screening methodology

- **Recommendation 1.2**: First-tier screening should measure 17-hydroxyprogesterone (17OHP) using immunoassays standardized on a common platform with gestational-age-specific reference ranges.
- **Technical Notes**:
    - Immunoassays remain common but may produce false positives.
    - Factors affecting results: prematurity, illness, early sample collection (<48h postnatal).
    - Repeat sampling and gestational age stratification to improve accuracy.

3. Second-tier Screening

- **Recommendation 1.3**: Laboratories should employ liquid chromatography–tandem mass spectrometry (LC-MS/MS) as a second-tier screen to improve specificity and positive predictive value.
- **Technical Notes**:
    - LC-MS/MS minimizes cross-reactivity seen in immunoassays.
    - Analysis of additional analytes, such as 21-deoxycortisol improves diagnostic precision.

4. Diagnostic Evaluation after positive newborn screening

- **Recommendation 3.1**: Infants with positive newborn screens should be referred promptly to pediatric endocrinologists.
- **Diagnostic Confirmation**:
    - Conduct serum testing for 17OHP, cortisol, and electrolytes.
    - Perform cosyntropin stimulation test if necessary.

5. Diagnostic Evaluation in Symptomatic Individuals Beyond Infancy

- **Recommendation 3.2**: Measure baseline early morning (before 8 AM) 17OHP by LC-MS/MS.
- **Recommendation 3.3**: If baseline 17OHP is borderline (200–1000 ng/dL), perform cosyntropin stimulation with full steroid profile (cortisol, 17OHP, androstenedione, 11-deoxycorticosterone, 11-deoxycortisol).
- **Recommendation 3.4**: Genotyping of CYP21A2 is suggested only when biochemical testing is equivocal or for genetic counseling.

7. Diagnostic Flowchart

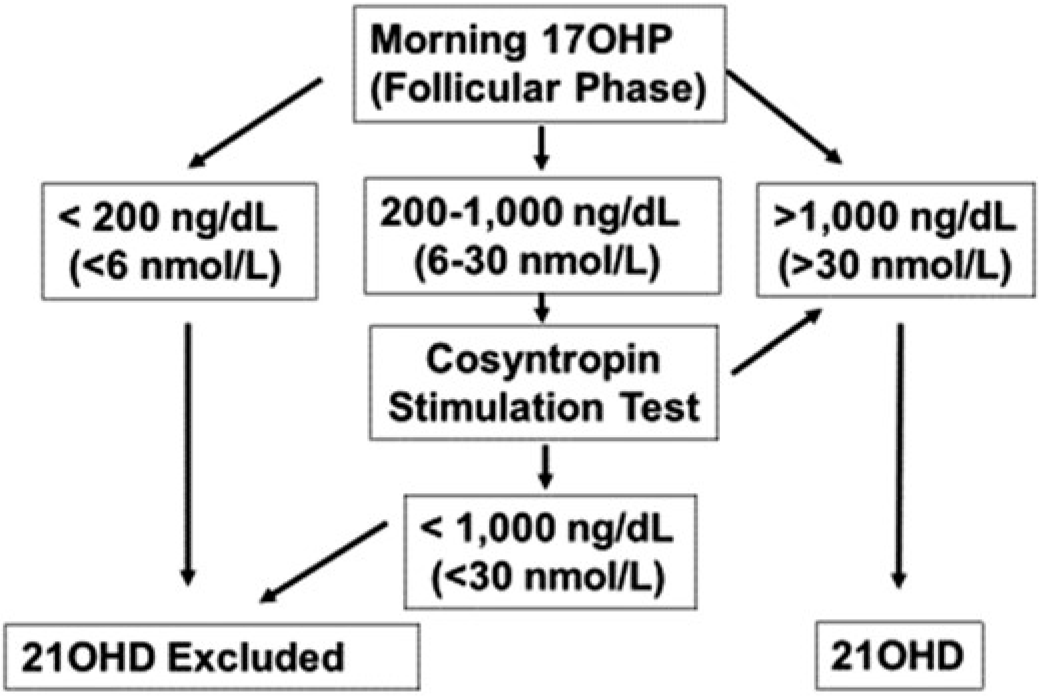

Figure S1. Diagnosis flowchart of 21OHD [1]**.**

1. Speiser, P.W.; Arlt, W.; Auchus, R.J.; Baskin, L.S.; Conway, G.S.; Merke, D.P.; Meyer-Bahlburg, H.F.L.; Miller, W.L.; Murad, M.H.; Oberfield, S.E.; et al. Congenital Adrenal Hyperplasia Due to Steroid 21-Hydroxylase Deficiency: An Endocrine Society* Clinical Practice Guideline. *J. Clin. Endocrinol. Metab.* **2018**, *103*, 4043–4088, doi:10.1210/jc.2018-01865.
